# Supplementary material for: Barriers and motivators for opioid deprescribing among Danish adults receiving long-term treatment with short-acting opioids: an exploratory qualitative study in primary care informed by self-determination theory
Source: Scand J Prim Health Care. 2026 Aug 26;44(1):2721337. doi: 10.1080/02813432.2026.2721337 (PMC13523111; doi:10.1080/02813432.2026.2721337)
Supplement: Supplementary file 1 [file IPRI_A_2721337_SM1362.docx]

**Title:**
Barriers and motivators for opioid deprescribing among Danish adults receiving long-term treatment with short-acting opioids: An exploratory qualitative study in primary care informed by self-determination theory.

**Authors:**
Sabrina Hoffensitz Nielsen^1^, shoffensitz@health.sdu.dk, https://orcid.org/0000-0002-0677-239X.

Line Bjørnskov Pedersen^1,2^, lib@health.sdu.dk, https://orcid.org/0000-0001-6804-2045.

Dorte Ejg Jarbøl^1^, DJarbol@health.sdu.dk, https://orcid.org/0000-0002-3241-419.

Merethe Kirstine Kousgaard Andersen^1^, mkandersen@health.sdu.dk, https://orcid.org/0000-0002-4833-1867.

Jens Søndergaard^1^, jsoendergaard@health.sdu.dk, https://orcid.org/0000-0002-1629-1864.

Carina Lundby^3,4^, carina.lundby.olesen@rsyd.dk, https://orcid.org/0000-0002-2121-6252.

**Affiliations:**
^1^ Research Unit of General Practice, Department of Public Health, University of Southern Denmark, Odense, Denmark.

^2^ DaCHE – Danish Centre for Health Economics, Department of Public Health, University of Southern Denmark, Odense, Denmark.

^3^ Hospital Pharmacy Funen, Odense University Hospital, Odense, Denmark.

^4^ Clinical Pharmacology, Pharmacy and Environmental Medicine, Department of Public Health, University of Southern Denmark, Odense, Denmark.

**Corresponding author:**

Sabrina Hoffensitz Nielsen.

ORCID: 0000-0002-0677-239X.

(+45) 60372730.

shoffensitz@health.sdu.dk.
Campusvej 55, 5230 Odense M, Denmark.

**Author contributions**

All authors contributed to the development of the study design. SHN generated all the empirical material and wrote the first draft of the manuscript. All authors contributed iteratively with adjustments and supplements to the manuscript, discussed the theoretical approach and the findings, and reviewed and approved the final version.

**Funding:**
Funding from the Fund for General Practice (grant no. A4840) is gratefully acknowledged. The sponsors were not involved in decisions regarding study design, data collection, analysis and interpretation of data, writing of the article, or decision to submit it for publication.

**Declaration og interest statement:**

Financial support was provided by Foundation for General Practice. Otherwise, the authors declare that they have no known competing financial interests or personal relationships that could have appeared to influence the work reported in this paper.

**Acknowledgements**

The authors want to thank all general practitioners who helped identify and reach out to patients. We also wish to give special thanks to all participants who shared thoughts and experiences on this sensitive topic. We would also like to thank participants at the Nordic Congress of General Practice 2026 for valuable input and discussions.

**Data availability**

There is no public availability of the interview transcripts outside of the research team due to confidentiality.

**Ethics**

The study’s data processing was registered with the Research & Innovation Organization at the University of Southern Denmark (file no. 11.409). The Regional Committees on Health Research Ethics for Southern Denmark reviewed the study and concluded that it did not require formal approval, in accordance with Section 14(1) of the Danish Act on Research Ethics Review of Health Research Projects (file no. 20212000-56). Collection and processing of personal data complied with Section 10 of the Danish Data Protection Act and Article 6(1)(e) of the EU General Data Protection Regulation (GDPR), permitting use of personal data for scientific purposes without written consent.

**Wordcount:**
Word count of article: 9031

Word count abstract: 250

**Barriers and motivators for opioid deprescribing among Danish adults receiving long-term treatment with short-acting opioids: An exploratory qualitative study in primary care informed by self-determination theory**

**Key messages:**

- We study patients’ barriers and motivators for opioid deprescribing in a qualitative study
- Three interlinked themes were generated from the patient narratives:
- Effects and consequences, significant relationships, and the work of identity
- Interpreted through a lens of self-determination theory, the findings suggest that:
- As-needed dosing can provide psychological reassurance enabling deprescribing
- Attentive and respectful care may foster more autonomous self-endorsed motivation for deprescribing

**Abstract**

**Background:** Long-term opioid treatment for chronic non-cancer pain (CNCP) yields limited benefit and notable risk of harm. Danish guidelines discourage long-term treatment with short-acting opioids, yet short-acting opioid prescribing persists.

**Aim:** To investigate the barriers and motivators to opioid deprescribing among adults with CNCP, receiving long-term treatment with short-acting opioids in primary care.

**Methods:** We conducted an explorative qualitative study using semi-structured, face-to-face interviews with adults with CNCP with long-term treatment with short-acting opioids invited through Danish primary care. The analysis was abductive inspired by reflexive thematic analysis using inductive coding and theme generation, followed by interpretation through the self-determination theory.

**Results:** 11 participants were interviewed (mean age of 62 years; eight women). Three themes were generated: 1) Effects and consequences, 2) Significant relationships, and 3) The work of identity. Participants experienced that as-needed dosing with short-acting opioid provided psychological reassurance that could enable tapering engagement. In line with self-determination theory, we found that the motivational quality towards medication change varied with the degree to which the needs for: autonomy, competence, and relatedness were satisfied. Attentive and respectful care fostered identified or integrated regulation, whereas suspicion promoted controlled motivation or amotivation. Ambivalence made tapering simultaneously desirable and untenable.

**Conclusions:** Sustainable opioid reductions among adults with CNCP who use short-acting opioids long-term is not merely a technical exercise. It is inherently relational and attentive to identity. Clinical practice could beneficially address ambivalence explicitly, rather than treating it as resistance, leverage short-acting opioid flexibility as reassurance, and support autonomy, competence, and relatedness.

**Keywords:** Long-term opioid treatment, short acting opioids, facilitators, motivators, deprescribing, primary care, self-determination theory

**Barriers and motivators for opioid deprescribing among Danish adults receiving long-term treatment with short-acting opioids: An exploratory qualitative study in primary care.**

# 1 Background

Chronic non-cancer pain (CNCP) is prevalent and difficult to manage for both patients and physicians (1), with a prevalence between 19-28% of the adult population (2, 3). Opioids have historically been widely used for pain relief, with short-acting opioids (e.g., immediate-release morphine) typically intended for acute pain or short-term titration (4, 5), and long-acting opioids (e.g., controlled-release morphine) used when opioid treatment is continued over time (4, 6). However, long-term opioid treatment for CNCP is increasingly questioned due to limited evidence of benefit and substantial risk of harm, including dependence, misuse, falls, fractures, cardiovascular events, and mortality (7-11)

Several countries have issued clinical guidelines that discourage LTOT for CNCP and advocate cautious deprescribing and use of non-pharmacological alternatives (4, 5, 12, 13). The Danish 2018 national guideline explicitly discourages long-term short-acting opioid use for CNCP (4). In 2023, close to 30% of adult Danes were estimated to live with CNCP (3), and approximately 7% of the population redeemed at least one opioid prescription (14). CNCP care is mainly provided by general practitioners (GPs), being the primary opioid prescribers (around 74% of initiations and 92% of maintenance) (15). According to a report from the Danish Health Data Authority, one third of long-term opioid users with chronic pain were treated exclusively with short-acting opioids in 2017 (16). Despite guidance, long-term short-acting opioid prescribing persists (17).

Evidence indicates that opioid tapering in primary care is not solely a technical process, but also a relational and ethical one (18-20). GPs must navigate guideline recommendations while addressing complex patient needs, often within the constraints of limited time, resources, and uncertainty regarding optimal tapering strategies (21-25). Existing qualitative studies show that patients often view opioids as medications of last resort that may be perceived as supporting quality of life, physical functioning, and coping with chronic pain (24, 26). Concerns about long-term adverse effects, medication-related harms, and quality of life may also prompt openness to deprescribing (27). At the same time, deprescribing may evoke fears of increased pain, withdrawal symptoms, reduced functioning, and disruption to established medication routines (24, 26, 27). Patient-clinician trust, credible information, and collaborative support have been identified as central to making deprescribing conversations acceptable and feasible (24, 26-28). Conversely, limited support, previous negative tapering experiences, and misalignment between patient and clinician perceptions of risk may hinder willingness to and engagement in deprescribing conversations (26, 27). When patients are motivated, perceive benefits, and experience collaborative support, tapering is reported to be more feasible (18-20, 29-31).

Importantly, motivation is not a unitary construct, and different motivation regulations may shape how patients engage with tapering efforts (32-34). Self-determination theory (SDT) offers a useful framework for understanding such motivational dynamics by distinguishing between more controlled and more autonomous forms of motivation (32-34). According to SDT, motivation is shaped by the extent to which three basic psychological needs are supported: autonomy, referring to the experience of volition and self-endorsement; competence, referring to a sense of capability and mastery; and relatedness, referring to feeling understood, respected, and supported by others (32-34). In healthcare contexts, autonomy-supportive and relationally responsive environments may facilitate the internalization of health-related behavior change, whereas controlling or need-frustrating encounters may contribute to resistance, ambivalence, or disengagement (18, 35-39).

Although barriers and motivators for opioid deprescribing are increasingly acknowledged (18, 22, 25, 38, 39), existing studies often address opioid use as a broad category (18, 22, 25, 38, 39). This may obscure formulation-specific dynamics that are relevant to deprescribing. The more immediate and situational use of short-acting opioids may become closely tied to patients’ everyday management of fluctuating symptoms, activity, and perceived control. Examining short-acting opioids specifically may therefore provide insight into motivational dynamics that are less visible when opioids are studied as a single overall category. In the Danish context, the specific attention to short-acting opioids in the national clinical guidelines, further supports its relevance. The aim of this study was to investigate the barriers and motivators for opioid deprescribing among patients with CNCP receiving long-term treatment with short-acting opioids in primary care.

# 2 Method

## 2.1 Study design

In this explorative qualitative study, we conducted semi-structured face-to-face interviews with adults living with CNCP and receiving long-term treatment with short-acting opioids in Danish primary care. The study was reported in accordance with the Consolidated Criteria for Reporting Qualitative Research (COREQ) (See appendix A) (40).

## 2.2 Participants and recruitment

Eligibility required adults (18 years or more) with CNCP, long-term treatment with short-acting opioids, no cancer diagnosis within the past five years, and the ability to take part in a one-hour interview. Long-term treatment with short-acting opioids was defined as repeated prescriptions of short-acting opioids over at least six months prior to recruitment, with ongoing use at the time of enrollment, aligning with Danish national guidance and opioid monitoring practices (4, 16, 41).

Participants were purposively sampled in collaboration with their GPs. GPs were invited via online announcements on the website for Network for Knowledge and Skills in General Practice (42) and in the Danish College of General Practitioners newsletter, as well as through direct email invitations to the research team’s GP network. Interested GPs received written information describing the study and patient eligibility criteria and were asked to 1) identify eligible patients in their practice, 2) provide them with written study information, and 3) obtain verbal consent to share patient contact details with the research team for further information about the project and participation.

While no strict minimum number of prescriptions was specified in the written inclusion criteria, GPs were informed verbally that our broader research program defines long-term opioid treatment as continuous redemption over at least six months, with no more than four months between prescriptions or between the last prescription and the end of the six-month period (43). This served as a guiding framework rather than a formal eligibility criterion, supporting the identification of patients with ongoing opioid use and supplementing the definition with clinical judgment.

For patients who consented, author SHN contacted each patient by telephone, provided verbal study information, and re-sent written materials when needed. Patients were encouraged to ask questions, and verbal consent was obtained before scheduling interviews. GPs received a standard fee of DKK 577 incl. VAT per enrolled patient (44).

## 2.3 Interview guide

The interview guide was developed by SHN in collaboration with an interdisciplinary research team comprising GPs (DEJ, MKA), a GP and clinical pharmacologist (JS), a pharmacist (CL), and a health economist (LBP). Its construction was informed by an exploratory review of qualitative and mixed-methods studies on chronic pain and opioid tapering (18, 19, 21, 31), supplemented by a key-informant interview with a physician from a specialized pain clinic in Denmark. The guide was iteratively refined within the research team to ensure clinical and policy relevance.

The final guide comprised open-ended questions addressing everyday life with chronic pain, experiences with opioid and non-opioid treatments, perceived benefits and harms of opioid use, knowledge of and concerns about side effects, experiences with or resistance to tapering, social and relational pressures, and expectations for future treatment (Appendix B). In line with the study’s exploratory orientation, the guide was used flexibly and adapted during interviews to incorporate issues raised by participants.

## 2.4 Data collection

Recruitment was conducted using purposive sampling and continued until sufficient information power was achieved (45), considering the study aim, sample specificity, use of theory, and the richness and relevance of the data.

Few participant characteristics (age and type of opioid treatment) were obtained verbally prior to the interview and audio recorded, while additional characteristics were collected during the interview. Gender was recorded based on the researcher’s observation and was not self-reported.

Interviews were conducted by SHN between February and August 2024. SHN holds a Master of Science in Medicine with industrial specialization, providing knowledge of basic medicine and health economics, but without a clinical specialization. This background allowed SHN to approach the interviews without the authority of a physician. Reflections on SHN’s role as researcher are presented in Appendix C.

All interviews were digitally audio-recorded, transcribed verbatim, and de-identified by SHN. Transcripts were not returned to participants for comments. Data was stored on a secure server and coded using NVivo (version 14) (46).

## 2.5 Data analysis

The study was informed by a social constructivist epistemological stance, acknowledging knowledge as socially and contextually situated (47, 48). The analysis was inspired by reflexive thematic analysis as described by Braun and Clarke (49, 50), rather than conducted as a strict application of the method. More specifically, the analysis drew on the recursive, researcher-active, and interpretive principles of reflexive thematic analysis, while adapting the approach to the aims of the present study. Specifically, the analysis combined inductive engagement with the empirical material and theoretically informed interpretation through SDT. In addition, themes were named in a relatively descriptive manner to enhance transparency and accessibility, rather than using more interpretive or conceptual theme labels. Thus, the analysis was abductive in nature, moving iteratively between the empirical material and theoretical interpretation.

The process was structured in two analytical phases, broadly corresponding to the six phases of reflexive thematic analysis. These phases were recursive rather than strictly sequential.

*Phase 1: Inductive coding and theme development (stages 1–4)*

All transcripts were read repeatedly to establish familiarity by author SHN. Three interviews were openly coded by SHN, and preliminary codes were discussed within the interdisciplinary research team to support reflexivity, challenge preliminary interpretations, and enrich the analytical process. The remaining interviews were coded iteratively by SHN, with codes refined, and reorganized as the researcher’s interpretation developed through the continued engagement with the data. Through this inductive process, patterns of meaning were identified and developed into preliminary themes by SHN.

*Phase 2: Theoretical interpretation (stages 5–6)*

In the second phase, the most salient themes were refined and interpreted through the lens of SDT (34) by SHN. The decision to use SDT was informed by the aim of the study. Given that SDT is a widely recognized theory of motivation (35, 37), it offered a robust and theoretically coherent analytical lens for examining the underlying motivational dynamics relevant to this context. While coding remained empirically grounded, this phase followed a more deductive orientation, using the theoretical framework to deepen interpretation of themes. SDT, which emphasizes the basic psychological needs of autonomy, competence, and relatedness, offered a useful perspective for understanding participants’ ambivalence, motivational conflicts, and perceived barriers to engage in deprescribing.

In this analytical phase, basic psychological need satisfaction was understood as shaping the quality of participants’ motivation for deprescribing. In line with SDT, motivation was interpreted along a continuum ranging from amotivation, where individuals lack intention or perceive change as futile, to controlled forms of regulation, including external regulation, where behavior is driven by external demands or pressures, and introjected regulation, where behavior is driven by internal pressures such as guilt, fear, or the need to avoid disapproval. More autonomous forms of regulation included identified regulation, where tapering is recognized as personally important, and integrated regulation, where reducing opioid use is experienced as aligned with broader values, identity, or long-term goals. Contexts supporting autonomy, competence, and relatedness were understood as facilitating more autonomous regulation, whereas need-frustrating experiences were interpreted as contributing to controlled regulation, ambivalence, or disengagement. This distinction guided the interpretation of how participants described their reasons for continuing, reducing, or resisting changes in short-acting opioid treatment. This two-phased abductive strategy ensured that the analysis was rooted in participants’ experiences while also drawing on theory to enhance interpretive depth. Lastly, the analysis was refined through discussions in the research team.

# 3 Results

A total of nine GPs from different general practices (located in Region Zealand, Region of Southern Denmark, and Central Denmark Region) identified eligible patients. 18 patients were contacted, of whom 12 consented to participate, representing six of the nine practices and two of the three regions (Region of Southern Denmark and Central Denmark Region). The primary cause of non-participation was no longer receiving treatment with short-acting opioids, as well as lack of time and energy. One patient had discontinued short-acting opioid treatment at the time of the interview and was excluded, resulting in 11 participants. Each interview lasted approximately one hour and took place in participants’ homes as preferred by participants, with no one else present during the interviews beside the participants and SHN.

All participants (mean age of 62 years; eight women) had complex medical histories with multiple pain conditions and long-standing opioid use (Table 1). All participants reported extensive contact with primary and secondary healthcare and had undergone repeated adjustments to opioid therapy, including changes in agent, formulations, and dose, and often across multiple prescribers, aiming to balance analgesia with adverse events and pain exacerbation. Most of the participants had experience with attempts to reduce their dosage, often described as iterative processes involving both reduction and subsequent dose increases in response to pain, adverse effects, and impacts on everyday functioning.

**Table 1 | Self-reported characteristics of participants (N=11)**

| Characteristic | | | Patients, No. (%) |
| --- | --- | --- | --- |
| Demographics | | | |
|  | Age, mean (SD) | | 62 (16) |
|  | Gender | |  |
|  | | Women | 8 (73%) |
|  | | Men | 3 (27%) |
| Chronic Pain Experience | | | |
|  | Primary pain condition | |  |
|  | | Musculoskeletal pain | 8 (73%) |
|  | | Neuropathic and other chronic pain | 3 (27%) |
|  | Duration of opioid therapy, years | |  |
|  | | >10 | 5 (45%) |
|  | | 2-5 | 6 (55%) |
|  | Type of opioid medication | |  |
|  | | Depot and as-needed | 7 (64%) |
|  | | As-needed | 4 (36%) |
|  | Opioid treatment initiated at | |  |
|  | | General practice | 5 (45%) |
|  | | Hospital | 6 (55%) |

*Table note: SD, standard deviation, No., number. Participant characteristics, including information on age, opioid medication type and use, were based on participants’ self-reports during the interviews.* *Gender was recorded based on the researcher’s observation and was not self-reported. Eligibility for participation was assessed by the participants’ GP according to predefined opioid use criteria.*

The themes are presented with relatively descriptive labels. These labels function as entry points into the inductively generated themes, while the interpretative work is developed within the thematic accounts and further elaborated through the theoretically informed interpretation using SDT.

Within patients situated narratives, three themes were generated informing how barriers for and motivation to engage in deprescribing are constructed: 1) Effects and consequences, 2) Significant relationships, and 3) The work of identity. Figure 1 gives an overview of the three main themes and the coherent subthemes.

**Figure 1 | Overview of the three main themes and coherent subthemes.**

## 3.1 Theme 1: Effects and consequences

Participants’ narratives highlight that perceptions of the effects and consequences of opioids are shaped through an interplay of knowledge, social expectations, and bodily experiences.

Within this theme three central subthemes were constructed from the analysis: ‘Everybody knows they are bad for you’ reflecting a socially embedded and widely shared knowledge perception of opioids as bad and harmful, ‘Pain relief at a price’ emphasizing the balance of pain relief and side effects, and ‘Enabling normality’ showing how opioids were perceived to enable sustainment of everyday functioning and social relationships.

### 3.1.1 Subtheme 1: Everybody knows they are bad for you

Across participants’ accounts, understandings of opioids were strongly shaped by information from health professionals and by negative media coverage (e.g., documentaries). Participants expressed a general view of opioids as harmful, unnatural, or even poisonous.

*“Well, I know it’s not good for the body to take that much. I mean. Everybody knows that.” (6)*

A fundamental skepticism toward opioids’ chemical influence was articulated through obvious assumptions resonating with broader societal discourses. Opioids were described as potentially damaging to organs and the body’s ’natural balance’. Some participants explicitly called opioids poison, while others worried that long-term use might affect inner organs. Others described opioids as unnatural and reported a moral as well as bodily resistance to taking them, indicating a moral corporeal resistance where opioids were constructed as foreign elements conflicting with an ideal of a natural, unpolluted body. Participants also voiced frustration about limited knowledge and research of long-term effects of opioids, which intensified uncertainty and vulnerability.

*“I think about my kidneys and my liver, and what opioids does do to the body? Yeah, in the long run. Yeah. And that’s also one of the reasons I’ve tapered down.” (11)*

Several moved from experiential claims to epistemic certainty. At the same time, participants relate to opioids as a necessary precondition for everyday functioning, which creates an inner conflict between need and concern.

*“And it’s not because I’m stupid and don’t know what it means, and what’s in it and that kind of thing. I do know that. I fully know the consequences of taking these things.” (10)*

### 3.1.2 Subtheme 2: Pain relief at a price

Overall, the accounts reflected a clear awareness that medication did not come without a price, and that pain relief had to be balanced against negative consequences. Several participants described that opioids ease the pain, enabling daily functioning, while emphasizing they do not eliminate pain. In addition, participants described a decreasing effect over time, leading to frustration being forced to increase dose. Also concern about this normalization of the opioids’ effect was mentioned.

*“… when I took them in the past, I could feel that I’d taken them. In my head, right? And now, when I take them now, I can’t feel it, and I even feel like I can drive. Not that I do…. And that scared me a bit. It really did.. that I could get used to it so fast, like, take it in, and that it could become so normal for me.” (7)*

Participants’ narratives showed that adverse effects were pervasive and a significant part of LTOT. These effects varied in type, intensity, and significance, shaping daily life and participants’ willingness to continue treatment. Fatigue, drowsiness, nausea, and emotional effects were particularly common. Reactions were experienced as more pronounced at initiation, e.g. dizziness, vomiting, or a sense of intoxication. Some described a euphoric effect, perceived as undesirable or unnatural. Participants also reported unwanted psychological effects, e.g. emotional distance, irritability, reduced libido, and behavioral changes. These effects were experienced as undermining relationships and affecting the perception of one’s identity.

*“I was always tired and had a very short fuse. No energy for anything. Nothing. You’re just tired, and you’re walking around in a ‘cheese-dome’ all the time, right? […] You are actually another version of your-self, when you get these types of medicine. You can’t be your-self 100%” (11)*

Adverse effects constituted a barrier to optimal pain relief, hence, several participants had reduced or switched opioid type in response. Nevertheless, adverse effects did not necessarily lead to discontinuation but entailed an ongoing balancing of analgesia against quality of life. Participants developed strategies to manage adverse effects, amongst others using short-acting opioids situationally, adjusting dosage, and modifying timing. These strategies illustrated considerable adaptation, competence, and self-care. The subtheme reflected a complex, ambivalent relation to opioids. Participants navigated between the desire for pain relief and fear of losing control, functional capacity, identity, or relational stability. There was clear awareness of physical and psychosocial consequences. Reflective use and developed personal tactics appeared to support the maintenance of balance. This reflexivity provides a solid foundation for working with participants’ own strategies.

*That’s why I try to take breaks now and then, precisely to sort of reset things a bit, because I don’t want to end up on too high a dose […] I’d like to get up to at least a week… […] I mean, if I’m aiming for a week, then of course I think about the following days, where I try not to plan anything. To make it easier for myself, to get through it. (1)*

A recurrent pattern was that complete pain relief was not perceived as ideal. It was rather framed as risky, potentially fostering dependence or an artificial way of living. Instead, participants emphasized being able to sense the body and preserving a manageable degree of pain as guidance and control. At the same time, they acknowledged that pain itself could be debilitating and that medication was therefore necessary, but only insofar as it enabled participation in everyday life without compromising consciousness, straining relationships, or undermining a sense of autonomy.

*“An acceptable level. You don’t have to be pain-free all the time, you know. […] Then you’re still aware that you have pain. You must never be lulled into a world where you’re totally pain-free, because then I think you also forget. And then I think you become even more addicted to opioids.” (1)*

### 3.1.3 Subtheme 3: Enabling normality

Opioids were experienced as essential to maintaining basic functioning and quality of life. Several described opioids making it possible to get out of bed, be physically active, and engage in social and family life.

*“I’ve gotten control back. I can use the opioids to get pain relief so I can function more like a human being. … So, if I take painkillers, I can go for a walk in the woods with my dog and my wife. Like, a short walk. Not far. … hmm. Sex, for example. I mean, important things that count as self-care…” (1)*

Some participants further described life without opioids as unbearable or meaningless and explicitly prioritized quality of life over longevity with pain. When opioids appeared in participants’ narratives as the only means of sustaining meaning and everyday function, alternatives were constructed as meaningless, hereby dissolving the very basis for imagining change. The perceived threat of losing medication foregrounded how possibilities for living meaningfully and maintaining social and personal roles were perceived as so constrained that motivation to taper weakened or vanished.

*“... because this weekend, when I’ve been in so much pain, I’ve been really glad that I had that as-needed medication. Otherwise, it would be almost unbearable to get through days like that and have so much pain.” (4)*

Some participants noted that short-acting opioids afforded need-based dosing, supporting quality of life, daily and social participation, and relief during acute exacerbations in contrast to long-acting opioid formulations with fixed dosing. These narratives highlighted a situated sense of control and flexibility in treatment within everyday contexts. The mere availability of short-acting opioids for as-needed use often functioned as psychological reassurance. Several described that simply having the medication at hand, even without daily intake, conferred security and control and sometimes emboldened attempts at tapering.

*“It gives… But you could call it a false sense of security. It gives a sense of security, at least, that I know I have it in the drawer if things get bad.” (5)*

## 3.2 Theme 2: Significant relationships

The participants’ narratives highlight that relationships play a crucial role in their experience with LTOT and motivation towards medication change. Two subthemes were constructed: ‘The significant relationship with healthcare professionals’ and ‘The significant network’. Together, these subthemes illustrate how both professional and personal relationships can act as key driving forces or barriers in participants’ change in opioid use.

### 3.2.1 Subtheme 1: The significant relationship with healthcare professionals

Trust between participants and physicians was constructed as a dynamic and relational process, continually negotiated in participants situated narratives of LTOT. It was not reducible to clinical expertise. Rather, it hinged on participants feeling recognized as whole people with unique histories, experiences, and values. When such recognition was enacted, participants described openings for dialogue and shared decision-making. These practices not only shaped participants’ sense of control and responsibility in treatment but also generated a broader feeling of being valued and affirmed.

*“But I’ve also been given the freedom to experiment with it. I mean, it’s not like I’m just allowed to do whatever, but they trust me enough to know I’m trying to find the best way for myself. But without developing a problematic use. ... So, the trust I have with my GP means everything to me.” (1)*

Trust emerged especially where physicians demonstrated attentiveness, respect, and continuity. Participants with long-standing relationships with GPs familiar with their histories constructed these encounters as being taken seriously and included in decision-making. Such interactions produced a sense of safety and fostered shared ownership of treatment. Such dynamics could be significant in a context marked by side effects, dependence concerns, and stigma.

Conversely, trust could easily be undermined, particularly in encounters with new physicians or where participants felt suspicion, standardized assessments without personal consideration, or a lack of time and attentiveness. In these situations, participants narrated having to defend their opioid use, which is described as degrading and burdensome. When GPs’ approaches were perceived as rigid or driven more by system logic than individual judgment, participants reported emotional distress and resistance within the relationship.

*“Since I got my GP, I’ve kind of been a bit dissatisfied with him. I didn’t really feel he took me seriously and kind of laughed a bit at, and.. when I had someone with me, it was that person who was being talked to, about me. But he didn’t talk with me, or to me. It’s only now we’re starting to have an okay conversation. Otherwise, I had actually been thinking of asking for another. He, he has been sarcastic...” (8)*

Trust was not static but continually (re)negotiated as treatment trajectories shifted, GPs changed, and participants’ life circumstances evolved. What mattered was not professional authority alone but how it was balanced with empathy and care, which shaped whether the relationship was experienced as trustworthy and supportive. In a treatment context where concerns about control and dependence were ever-present, trust was not merely a relational quality but a precondition for experiencing care as meaningful and sustainable. When participants felt pressure from professionals, these relationships shaped how pressure was interpreted. Requests to deprescribe, when communicated respectfully and empathetically, invited reflection and, in some cases, led to substantial reductions in use.

*“... because she [physician at hospital] was, like, totally blown away that I’d just done it [reduced use] after so many years, right. And I got kind of stubborn, and I just thought ‘that, I can totally do it, I have to. Now I’m going to see how far I can actually get down [reduce], instead of walking around filling my body with this.’ I think she’s the one who has had the most, influence in this, to.. Yeah.” (7)*

*“But she’s [GP] also good at praising the other way around. [Has that helped?] Yeah, yeah yeah. Because then I was also kind of like, I will show them that I can [reduce].” (7)*

Conversely, suggestions or remarks perceived as sarcastic, condescending, or relationally distant were met with anger and resistance. Especially where the patient–physician relationship was fragile, pressure was narrated as mistrust rather than support, undermining motivation for change. This tension suggested that it was not the request itself but the mode of communication and the relational context that determined whether participants interpreted pressure as constructive or destructive.

### 3.2.2 Subtheme 2: The significant network

Support from close relationships played an important role in how participants managed LTOT and their motivation to change use, serving as emotional anchors and as practical or structural supports. Partners’ and children’s understanding, engagement, and concern often strengthened participants’ motivation to take responsibility for and maintain control of or change their opioid use.

*“I mean, I want to be with her [wife] and show that I can control this, and I don’t want to end up somewhere I don’t want to be and lose everything I’ve fought so hard for. So, she’s a huge motivator now. Now my son is too. Yeah, so that’s a motivator to constantly keep on top of my intake and make sure it never becomes uncontrollable.” (1)*

At the same time, support did not necessarily entail openness. Several participants practiced selective disclosure to protect themselves or relatives from stigma and worry. Comments from family members figured less as explicit demands or pressure and more as expressions of concern. Participants often acknowledged the intent, yet some questioned their validity, emphasizing that relatives could not fully understand their pain. This rejection illustrated how participants drew boundaries in relation to close networks, and how the family’s voice did not necessarily challenge or alter medication use. Instead, it registered as a background echo of concern, reinforcing a sense of being alone with both pain and medication decisions.

## 3.3 Theme 3: The work of identity

Participants’ narratives revealed that long-term treatment with short-acting opioids shaped not only their bodies and everyday lives, but also their sense of identity. The analysis identified two interrelated themes that mutually influence each and together constitute the identity-shaping factors in this material: ‘Personal identity and self-perception’ and ‘Societal identity and stigma’. These dimensions mutually influence each other and highlight how identity work is embedded in experiences with long-term opioid therapy, shaping participants’ ambivalence and their motivation to continue, reduce, or discontinue medication use.

### 3.3.1 Subtheme 1: Personal identity and self-perception

Participants’ narratives demonstrated that long-term treatment with short-acting opioids was entangled with complex, often ambivalent self-understandings. A recurrent tension surfaced between the need for pain relief and the desire to preserve control, dignity, and autonomy. Some drew on prior episodes of overuse or fears of losing control which shaped how they related both to the medication and to themselves as users. This ambivalence was especially evident in participant developed strategies to preserve responsibility and agency e.g., reducing doses, avoiding routine intake, taking pauses, or storing tablets out of reach. These practices could be read both as doubt about one’s own judgment and as sources of pride and self-respect, as they emphasized participants’ capacity to make active choices and to manage on their own.

*“I don’t want to be dependent on anything or anyone, and at the same time I like to have the control myself. So, I’m also a little bit proud that I could taper it off myself and say, now I have. I have chosen. No one has chosen for me that I don’t take daily medication. And I know I don’t get any carrot for that, but I feel good about having made an active choice.” (5)*

Participants emphasized presenting themselves as strong and capable rather than as victims. They wanted to be seen as people living with pain, not as weak or dependent. This effort was linked to the experience that, although pain is inherently subjective and not directly observable to others, their pain was often not accompanied by visible signs or socially recognizable markers of injury or illness. As a result, they felt compelled to continually legitimize or justify their pain to others. Such demands were experienced as exhausting and hurtful, yet participants noted that they, due to their situation, had also fostered greater self-awareness and increased empathy toward others in similar situations.

Taken together, the accounts suggested that opioid use and deprescribing was not merely a medical practice but a central site of ongoing identity work. Participants continually negotiated positions between being ill and strong and being dependent and responsible. For some, this entailed a rupture with prior self-understandings (e.g., as independent or professional). For others, it opened space for new insights and active efforts to preserve a dignified, positive sense of feeling proud of self within a complex social context.

### 3.3.2 Subtheme 2: Social identity and stigma

Participants’ narratives demonstrated that using short-acting opioids was not merely a medical practice but a socially and culturally charged act in which stigma played a central role. Participants described being met with assumptions or remarks from healthcare professionals, family members, and even strangers experienced as offensive, misconstruing, or directly hurtful.

A central element in this identity work concerned how participants positioned themselves in relation to the label addict. Participants invoked stereotypical images of the ‘real addict’ to positioning themselves in contrast. These depictions functioned as foils to participants’ self-understandings as responsible and functional and thus not addicts. Several acknowledged episodes of exceeding prescribed use yet underscored that such episodes did not count as addiction.

*“I’m not a drug addict. Far from it. I’m just. I just have chronic pain and poor health. So, I feel. I mean. I don’t feel like a drug addict. Not at all.” (11)*

When used by others, the labels addict or luxury addict elicited strong reactions, provoking shame, anger, and worthlessness. In addition, participants drew a clear boundary between physician-prescribed, legitimate use and illegal or uncontrolled consumption. This linguistic and behavioral distancing functioned as a key defense against the identity threat of being perceived as, or constructed as, an addict. Participants reported that certain opioids e.g., methadone or fentanyl were strongly linked with stigmatizing images of illicit or addictive use rather than legitimate pain management. When healthcare professionals used the label, it was experienced as deeply offensive and undermining trust. To avoid condemnation, some participants concealed their treatment. Others timed their medication around social activities to minimize visible side effects that might provoke suspicion. In this way, participants enacted ongoing efforts to protect relationships from stigmatizing associations. Stigma was not confined to close relations. Several pointed to public debate and policy measures on opioids as an abstract yet potent pressure. Participants expressed concern that wide policy restrictions would also affect those who used the medication responsibly. This societal pressure evoked fears of being categorized with addicts and was experienced as a threat to the legitimacy of their treatment. For some, it settled into a diffuse threat that generated discouragement and everyday uncertainty.

*“I get so scared when they talk about wanting to take it away, and the new thing that’s come now with… our Minister of Health talking about it so… yeah. I don’t know what the alternative would be. I mean, I really fear it. Yeah. But I also understand why it’s necessary. I’m just afraid they’ll lump too many together. […] But I don’t think they think about what it gives. Some people who actually have it under control.” (1)*

At the same time, responses to stigma varied considerably. Some expressed indifference to others’ perceptions, anchored in close relationships where understanding felt greater. In addition, openness was framed as a strategy to counter prejudice for both oneself and for others in similar situations.

Participants’ narratives showed that stigma manifested interpersonally, within healthcare practices, and societally through policy and media discourses. For some, opioid use became an identity burden requiring continual work to preserve dignity. For others, it was an accepted aspect of living with chronic pain that needed no explanation. Across these differences, a shared perspective was that they would not want to be seen as stereotypes and wanted to maintain their dignity and social position.

## 3.4 Interpretation using SDT as analytic lens

Through an SDT lens, the three identified themes, effects and consequences, significant relationships, and the work of identity, demonstrate how barriers and motivators are shaped through discourses, pain experiences, relationships, and identity negotiations by shifting configurations of need satisfaction and need frustration.

### 3.4.1 Autonomy

Within SDT, autonomy is understood as people’s need to self-regulate their experiences and actions. Their behaviors are self-endorsed and align with their own interests and values (34). Across participants’ narratives, autonomy appeared both supported and undermined. Resistance to ‘toxic’ or ‘unnatural’ opioids expressed a desire to live in line with values of health, purity, and bodily control, which for some participants motivated attempts at dose reduction or discontinuation, yet persistent pain often left no viable alternative, constraining autonomy in relation to deprescribing decisions. In contrast, maintaining lower doses or regaining normality through pain relief strengthened autonomy by aligning opioid use with personal goals and everyday functioning, illustrating how partial deprescribing could support a sense of self-determination when aligned with participants’ priorities. Clinical encounters played a crucial role: trustful relationships with GPs enabled shared decision-making and ownership of treatment, so that tapering or adjustments were not experienced as imposed but as resonating with participants’ own values, thereby supporting autonomy in deprescribing processes. Family life also shaped autonomy. Selective disclosure of opioid treatment allowed participants to protect themselves and their loved ones from stigma while preserving control over medication decisions, illustrating how autonomy and relatedness could be maintained simultaneously, even if this required careful management of what was shared. Finally, identity work was central: efforts to sustain a responsible self-image, such as reducing dosage or distancing from the addict label, safeguarded autonomy in the face of stigma, and could thereby reinforce willingness to engage in deprescribing. Yet this balance was fragile. When valued self-understandings, such as being independent or professional, clashed with the position of being a chronic pain patient receiving opioid treatment, autonomy was threatened, producing a diminished sense of ownership in one’s own life. In addition, as-needed intake supports autonomy as it enables choosing when and whether to take medication, which could also support engagement in gradual deprescribing strategies.

### 3.4.2 Competence

Within SDT, competence refers to people’s basic need to feel capable and confident. They want to feel able to handle things well in the parts of life that matter to them (34). Participants’ narratives revealed both support and frustration of this need. Opioid treatment could restore functionality and enable meaningful activities, thereby reinforcing competence and sustaining motivation for continued use. Yet uncertainty about long-term effects, the emergence of tolerance, and disruptive side effects often undermined feelings of self-efficacy over managing pain and navigating treatment, leaving participants insecure and powerless or uncertain about how to act, which could prompt reconsideration of their current treatment and open for motivation towards change. Clinical relationships played an important role in shaping competence. Continuity and trust in professional expertise offered reassurance and helped participants navigate a complex treatment landscape, whereas skepticism from healthcare professionals weakened legitimacy and self-efficacy, fostering amotivation for change, particularly when participants’ sense of mastery over their pain and treatment was already fragile. Similarly, family pressure to reduce opioids generated discouragement or even amotivation, if the participant wasn’t confident in one’s ability to do so. Conversely, when opioid reduction was experienced as aligned with values such as responsibility, control, and dignity, participants described enhanced competence and greater likelihood of internalizing change as meaningful and self-endorsed. Notably, as-needed use also supports competence by reducing fear of uncontrollable pain and strengthening capacity to handle future challenges. The mere availability can be sufficient. Having a safety net at hand produces calmness via the assurance of possibility and being able to refrain while knowing the option exists becomes an active strategy that sustains mastery and control.

### 3.4.3 Relatedness

Within SDT, relatedness refers to feeling socially connected, having a sense of belonging and feeling valued by others. It also includes experiencing oneself as someone who gives to or contributes to others (34). Participants’ narratives highlighted how opioid use affected this need in ambivalent ways. On the one hand, preserving intimacy, family life, and social participation motivated efforts to maintain lower dosages or engage in deprescribing, as adverse effects were seen to threaten belonging. At the same time, when pain relief enabled normality and sustained social roles, opioid use supported relatedness by safeguarding meaningful connections which could in turn reduce motivation to change.

Clinical relationships also played a central role. Attentiveness, respect, and continuity fostered recognition of participants as whole persons and thereby supported internalization. This process made participants more receptive to deprescribing requests, allowing treatment decisions to be experienced as meaningful rather than imposed. Conversely, relational distance, sarcasm, or distrust undermined relatedness and left participants to defend themselves. In this context, deprescribing requests were less likely to be internalized and more often perceived as imposed, eroding recognition of participants as active agents in their own care. In families, care and concern provided relational support that anchored stable and controlled use or motivation to reduce opioid consumption. However, lack of experience-based understanding could partially frustrate relatedness and limit the acceptance of support. Being recognized as a person in pain rather than as an addict was crucial for sustaining relatedness, whereas mistrust, labeling, or distance undermined this need and hindered motivation for change. In response, participants deployed relational strategies such as withholding use or reframing medication to protect relations. Others resisted stigma through openness or by distancing themselves from external judgments, demonstrating how relational defenses could buffer frustration and, in some cases, enable a more autonomous engagement with opioid use.

### 3.4.4 Regulation and amotivation

The first identified theme, ‘Effects and consequences’, shows that shifts from external regulation toward more self-determined forms such as identified regulation can take place when actions align with participants’ values and goals even if not inherently enjoyable. Dosage and use decisions can thus reflect internalized motivation anchored in values, relationships, and a need for control rather than in external pressures or pain alone. When life without opioids is described as meaningless or unbearable, this indicates amotivation toward deprescribing. According to SDT, amotivation may arise when behaviors lack meaning or when change threatens basic needs, such as when people resist demands that undermine their basic need for autonomy or relatedness (32-34, 36, 37). Participants particularly point to the latter, since opioids are closely tied to acting more freely and sustaining relationships. In parallel, the ambivalence of opioids being perceived as both toxic and life-improving points toward introjected regulation. That means use accompanied by guilt or moral conflict rather than fully integrated acceptance (32, 37).

The second identified theme, ‘Significant relationships’, shows that the quality of relationships matters. Respectful, empathetic framing enabled shifts away from controlled regulation toward identified regulation, where tapering could be experienced as meaningful and self-endorsed. By contrast, controlling communication such as sarcasm and relational distance, diminishes feelings of relatedness, yielding more controlled regulation or amotivation (32-34, 36, 37, 51). Two amotivation routes were evident: 1) defiance when demands were experienced as thwarting autonomy and relatedness, and 2) inefficacy when expectations exceeded perceived competences (32, 33, 36, 37).

The third identified theme, ‘The work of identity’, shows that opioid use can be regulated through identified values such as responsibility and control, supporting more autonomous motivation. Threats to the self-image can, on the other hand, lead to resignation or passivity, with use no longer experienced as self-chosen but as something to which one is subjected. Stigmatization further threatens autonomy. Feeling reduced to a negative category, or experiencing political restrictions and public debate as controlling, can shift regulation from an autonomous basis toward controlled motivation and, at times, amotivation when behavior is felt neither meaningful nor possible (32, 33, 36, 37, 52). Anger and resistance when labeled as addicts can be read as amotivation where defiance is used to protect autonomy. At a societal level, diffuse pressure from debate and restrictive policies increases the risk of movement from autonomous regulation toward controlled forms, and in worst cases into discouragement and powerlessness (32, 33, 36).

### 3.4.5 Synthesis

The contrasting constructions of opioids as both toxic and life-improving situate sense making within a tension between concern and necessity. Clinical encounters that acknowledge concern and dilemmas while strengthening competence and autonomy may support a shift in motivation from guilt-driven compliance toward more internalized, self-endorsed engagement with reduction.

Across clinical and familial contexts, trust, respectful communication, continuity, and value-congruent framing support autonomy and relatedness. It may also indirectly support competence, enabling movement toward identified regulation. Sarcasm, condescension, relational distance, and mis-attuned pressure frustrate needs. This could contribute to controlled regulation or amotivation.

When participants feel reduced to stigmatized categories, or when mistrust and policy are experienced as external control, autonomy and relatedness might be undermined, which could foster controlled motivation or resignation. By contrast, relationships characterized by respect, trust, and understanding more readily support autonomy, competence, and relatedness, which may allow reduction or change in opioid treatment to be experienced as a self-chosen, value-based step. Across the analysis, self-image is not merely reflective but an active resource as it can hinder or support motivation to regulate and potentially reduce opioid use.

# 4 Discussion

This study examines barriers and motivators for opioid deprescribing among Danish CNCP adults receiving long-term short-acting opioid treatment. Three themes were generated. First, effects and consequences, second, the significant relationships, and third, the work of identity. Decisions about opioid use are socially situated practices interwoven with sense making, relationships, and identity, hence, not reducible to biomedical balancing of pain relief and side-effects.

This supports and extends previous qualitative literature showing opioids as simultaneously enabling and burdensome, and that deprescribing is shaped not only by clinical indicators and risk assessment, but also patients’ lived experiences of pain, fears of deterioration, trust in clinicians, and perceived legitimacy as pain patients (18-22, 24, 25, 31, 38, 39). By focusing specifically on participants’ experiences with short-acting opioids, this study adds a formulation-sensitive perspective to the literature on opioid deprescribing. Current guidelines generally discourage LTOT for CNCP and recommend optimizing non-pharmacological and non-opioid pharmacological treatments before considering opioids. Where opioids are considered, this should be understood as an individualized and closely monitored trial in selected patients, with attention to both potential benefits and harms (4, 5, 53).

Against this background, our findings should not be read as an argument for short-acting opioids as a preferred treatment strategy for CNCP. Rather, they show how participants perceived the flexibility of as-needed medication as both practically useful and psychologically reassuring. Flexible short-acting opioid treatment functioned not only as a treatment strategy in participants’ accounts, but also as a motivational resource, opening tapering possibilities by allowing experimentation while retaining a perceived safety net. This contrasts with some of the participants’ experiences of long-acting opioids, where fixed schedules felt more controlling and less adaptable to fluctuating pain. In this respect, our findings extend previous work on patients’ fears and concerns of tapering and loss of pain control by showing how the formulation itself may become part of the perceived safety structure around opioid treatment (18, 20, 38, 39). Distinguishing between formulations is therefore important, not to support LTOT, but to understand patients’ perspectives and to design deprescribing interventions that are responsive to the perceptions, fears and concerns, and practical needs attached to different medication forms. In this context, although self-management has been highlighted as central in deprescribing literature (27), it was not generated as a distinct theme in our analysis. Rather, elements of self-management were embedded across participants’ accounts of flexible medication use, their ongoing negotiation of treatment effects, and their work of identity. This suggests that self-management in our data is not experienced as a bounded individual capacity, but as a context-dependent and relational practice that cuts across themes.

Ambivalence was pervasive. Participants drew on bodily experiences and broader discourses to construct opioids as poisonous, unnatural, or morally suspect, while also describing them as indispensable for everyday participation. This ambivalence has been described in earlier work (38) and is also reflected in qualitative evidence syntheses showing that people using opioids for chronic pain often negotiate competing concerns about pain relief, dependence, stigma, withdrawal, and loss of function (22, 39). Yet our analysis shows how it directly shapes motivation through tensions between psychological needs. Autonomy was thwarted when participants felt compelled to act against values of health and purity, competence was undermined by uncertainty about long-term risks, adverse effects and tolerance. At the same time, opioids often supported feelings of competence and relatedness by enabling daily functioning and relationships. Motivation to continue often remained at the level of introjected regulation, driven by guilt, toxicity fears, or external expectations, helping explain why tapering felt both desirable and untenable. Clinical encounters could therefore beneficially address ambivalence explicitly, for example by exploring the concerns, values, and experiences underlying patients’ ambivalent feelings about opioid use and reduction, rather than treating ambivalence as resistance.

Relational contexts appeared to play an important role in whether motivational tensions were reinforced or alleviated. Trusting GP relationships, marked by attentiveness, continuity, and shared decision-making, supported autonomy and relatedness, made tapering requests feel supportive rather than coercive. Encounters characterized by suspicion or condescension undermined trust and could shift regulation toward controlled motivation or amotivation. Communication style thus appeared to shape motivation directly by influencing whether patients experienced tapering discussions as supportive and collaborative or as externally imposed and threatening, consistent with prior research highlighting how patients’ trust and engagement in opioid tapering are closely tied to how such conversations are conducted (20). This also aligns with studies of opioid management in primary care showing that guideline-concordant opioid reduction is difficult when conversations are experienced as abrupt, mistrustful, or insufficiently responsive to patients’ fears about pain and functioning (19, 21, 23-25, 38), highlighting the importance of trust in chronic pain management. Notably, short-acting opioid flexibility could be mobilized as a relational resource, and having medication available provided reassurance even with limited intake. For GPs, acknowledging and strategically engaging this safety net can sustain trust and protect relatedness during tapering conversations, presenting reduction as a process that fosters more autonomous and self-endorsed motivation for change.

Social networks were sources of both support and tension. Family care could strengthen motivation to use opioids responsibly, anchoring behavior in relatedness and responsibility. This finding extends previous work describing family support as both supportive and pressuring (38), by showing how relatives’ concerns may become difficult to receive when perceived as lacking experiential legitimacy or embodied understanding of chronic pain. In such situations, concerns could be experienced less as support and more as interference, producing feeling of isolation. This is consistent with research on feeling misunderstood in opioid tapering (39, 54). Such dynamics underscore how the need for relatedness can be simultaneously a motivator and a source of frustration. Interventions that involve families may therefore need to balance acknowledgment of relatives’ concerns with careful protection of participants’ autonomy and dignity.

Finally, LTOT and changes in use were also entangled with identity work. Participants sought to sustain self-images of responsibility and strength while distancing themselves from stigmatized categories such as addict or junkie. This dynamic resonates with sociological accounts showing that people prescribed opioids for chronic pain actively distinguish themselves from stigmatized drug user identities to preserve a sense of legitimacy and moral worth (55, 56). Our findings demonstrate how stigmatization undermines all three psychological needs. Autonomy is undermined by imposing externally defined labels, competence by eroding participants’ sense of legitimacy, and relatedness by threatening belonging. When opioid reduction was framed in ways that aligned with participants’ identity work and values, such as responsibility and control, it was more likely to be experienced as self-endorsed. When framed through stigmatizing lenses, participants reported resistance or resignation. Interventions should recognize and affirm participants’ identity strategies such as dosage control or selective disclosure rather than pathologize them. Narrative approaches and value-based therapies (e.g., acceptance and commitment therapy) may help align deprescribing with identity work (57, 58).

## 4.1 Strengths and limitations

This study has several limitations as well as strengths. First, the definition of long-term treatment with short-acting opioids used in this study was relatively broad, aiming to capture a range of sustained opioid use patterns relevant to the study aim and reflecting Danish clinical guidelines and monitoring practices (4, 16, 41, 43). While this enhances relevance to real-world clinical contexts, it also results in a more heterogeneous sample, which may shape how the findings are interpreted and their transferability to more specific patient groups.

Recruitment through GPs introduced an element of gatekeeping, as it is reasonable to assume that the GPs approached participants perceived as relatively stable and articulate, as this is also aligned with the inclusion criteria. This means that our sample may not capture perspectives from people living with severe dependence, unstable life circumstances, or limited capacity to participate. Certain perspectives may therefore not have been captured or might have been more pronounced in a different sample. This particularly holds for participants at the margins of misuse and social vulnerability.

Another limitation concerns the depth and richness of the data. Our insights derive from 11 interviews, which provided valuable access to participants’ reflections and sense making, but did not allow us to observe their interactions with healthcare professionals, family, or workplace. More prolonged engagement or ethnographic fieldwork might have generated richer and more contextualized material, providing insight into everyday negotiations around opioid use that remain less visible here.

A further limitation is the lack of patient and public involvement in the development of the interview guide. Although the guide was iteratively adapted during data collection to reflect participants’ perspectives, early involvement of patients may have strengthened its relevance and sensitivity.

Also, participant characteristics, including information on opioid medication type and use, were based on participants’ self-reports during the interviews. Consequently, some medication details, particularly current dosage or exact frequency of use, may be imprecise. However, participants were identified as eligible by their GP according to predefined opioid use criteria, reducing the risk of incorrect inclusion based solely on self-report. In addition, the focus of the study was not the pharmacological accuracy of opioid use, but participants’ barriers and motivators for opioid deprescribing. From this perspective, participants’ accounts of their medication use were analytically relevant as part of how they made sense of treatment, dependency, pain management, and possible tapering. Thus, while self-reported medication information should be interpreted with caution, it does not undermine the study findings.

At the same time, a key strength of the study is our focus on short-acting opioids. Distinguishing them revealed features otherwise overlooked, notably dosing flexibility and the psychological reassurance participants described. Another strength is our use of SDT as an interpretive framework, which moved us beyond description to analytic insights into how motivation is shaped through the interplay of bodily experiences, social relationships, and identity work.

# 5 Conclusion

The present study investigated barriers and motivators for opioid deprescribing among patients with CNCP receiving long-term treatment with short-acting opioids in primary care. Short-acting opioids were seen as both a problematic necessity and a valued resource as they relieve pain but also provoke fear and concerns, stigma, and threats to identity. While experiences such as side effects, tolerance, and perceived harm may support more controlled forms of motivation for tapering, supportive, trust-based relationships, non-stigmatizing, identity-preserving communication, and reassurance strategies (such as flexible, as-needed use) may support a shift towards more autonomous, self-endorsed forms of motivation (e.g., identified and integrated regulation) for deprescribing.

In practice, deprescribing may be better supported when ambivalence is addressed directly, short-acting opioids are framed as a safety net, and reduction is approached as a relational, identity-sensitive process. Such strategies may support patients’ basic psychological needs and foster more autonomous self-endorsed motivation towards changes in opioid treatment. In summary, sustainable reduction in opioid treatment appears to require attention not only to pharmacology and pain management but also to ambivalence, relational contexts, and identity negotiations.

# 6 References

1.	Rufener L, Akre C, Rodondi P-Y, Dubois J. Management of chronic non-cancer pain by primary care physicians: A qualitative study. PLoS One. 2024 Jul 26;19(7). https://doi.org/10.1371/journal.pone.0307701

2.	Reid K, Harker J, Bala M, Truyers C, Kellen E, Bekkering G, et al. Epidemiology of chronic non-cancer pain in Europe: narrative review of prevalence, pain treatments and pain impact. Curr Med Res Opin. 2011 Feb;27(2). https://doi.org/10.1185/03007995.2010.545813

3.	Ekholm O, Herling S, Lykke C, Skurtveit S, Hamina A, Sjøgren P, et al. Monitoring Chronic Non-Cancer Pain in Denmark Over Two Decades: Prevalence, Mental Health and Loneliness. Eur J Pain. 2025 Feb;29(2). https://doi.org/10.1002/ejp.4776

4.	Sundhedsdatastyrelsen. National Klinisk Retningslinje for Opioidbehandling af Kroniske Non-Maligne Smerter [Internet]. 2019 [cited 2025 Jun 19]. Available from: https://www.sst.dk/-/media/Udgivelser/2018/NKR-opioid/NKR-opioidbehandling-kroniske-non-maligne-smerter-endelig.ashx?sc_lang=da&hash=959340F77FA955F6B9F11F5C55392621.

5.	Dowell D, Ragan KR, Jones CM, Baldwin GT, Chou R. CDC Clinical Practice Guideline for Prescribing Opioids for Pain - United States, 2022. MMWR Recomm Rep. 2022;71(No. RR-3):1–95. https://doi.org/10.15585/mmwr.rr7103a1

6.	Argoff CE, Silvershein DI. A Comparison of Long- and Short-Acting Opioids for the Treatment of Chronic Noncancer Pain: Tailoring Therapy to Meet Patient Needs. Mayo Clin Proc. 2009 Jul;84(7). https://doi.org/10.1016/S0025-6196(11)60749-0

7.	Birke H, Ekholm O, Sjøgren P, Kurita G, Højsted J. Long-term opioid therapy in Denmark: A disappointing journey. Eur J Pain. 2017 Oct;21(9). https://doi.org/10.1002/ejp.1053

8.	Chou R, Deyo R, Devine B, Hansen R, Sullivan S, Jarvik J, et al. The Effectiveness and Risks of Long-Term Opioid Treatment of Chronic Pain. Evidence report/technology assessment. 2014 Sep(218):1–219. https://doi.org/10.23970/AHRQEPCERTA218

9.	Chou R, Hartung D, Turner J, Blazina I, Chan B, Levander X, et al. Opioid Treatments for Chronic Pain. [Interrnet]. Rockville (MD): Agency for Healthcare Research and Quality (US); 2020 Apr. Report No.: 20-EHC011. Available from: https://pubmed.ncbi.nlm.nih.gov/32338848/.

10.	Foster N, Anema J, Cherkin D, Chou R, Cohen S, Gross D, et al. Prevention and treatment of low back pain: evidence, challenges, and promising directions - PubMed. Lancet (London, England). 06/09/2018;391(10137). https://doi.org/10.1016/S0140-6736(18)30489-6

11.	Tucker H, Scaff K, McCloud T, Carlomagno K, Daly K, Garcia A, et al. Harms and benefits of opioids for management of non-surgical acute and chronic low back pain: a systematic review. Br J Sports Med. 2020 Jun;54(11). https://doi.org/10.1136/bjsports-2018-099805

12.	Busse J, Craigie S, Juurlink D, Buckley D, Wang L, Couban R, et al. Guideline for opioid therapy and chronic noncancer pain. Canadian Medical Association journal. 05/08/2017;189(18). https://doi.org/10.1503/cmaj.170363

13.	Häuser W, Schug S, Furlan AD. The opioid epidemic and national guidelines for opioid therapy for chronic noncancer pain: a perspective from different continents. Pain Reports. 2017;2(3):e599.

14.	Sundhedsdatastyrelsen. Langtidsbrug af opioider på recept falder. [Internet]. The Danish Health Data Authority; 2025. Available from: https://sundhedsdatastyrelsen.dk/Media/638784030005472714/Langtidsbrug_af_opioider_2018-2023.pdf#:~:text=Knap%20400.000%20personer%20har%20indl%C3%B8st%20recept%20p%C3%A5%20opioider,ca.%20145.000%20personer%20i%202023%2C%20jf.%20Figur%201.

15.	Pottegård A, Sørensen A, Olesen M, Rasmussen L. Opioid prescriber responsibility: A Danish drug utilization study British Journal of Clinical Pharmacology. 2023;89(4):1425–30. https://doi.org/10.1111/bcp.15595

16.	Sundhedsdatastyrelsen. Medicinforbrug - Indblik. Færre langtidsbrugere af opioider i 2017. Sundhedsdatastyrelsen.dk: The Danish Health Data Authority; 2018. Available from: https://sundhedsdatastyrelsen.dk/media/16184/MedicinForbrug_Indblik_Opioider_2017.pdf.

17.	Sundhedsdatastyrelsen. Statistik for lægemiddelsalg i Danmark [Internet]. MEDSTAT.DK; 2025 [cited 2025 24. Sep.]. Available from: https://medstat.dk/.

18.	Frank JW, Levy C, Matlock DD, Calcaterra SL, Mueller SR, Koester S, et al. Patients’ Perspectives on Tapering of Chronic Opioid Therapy: A Qualitative Study. Pain Medicine. 2016;17(10):1838–47. https://doi.org/10.1093/pm/pnw078

19.	Kennedy L, Binswanger I, Mueller S, Levy C, Matlock D, Calcaterra S, et al. "Those Conversations in My Experience Don't Go Well": A Qualitative Study of Primary Care Provider Experiences Tapering Long-term Opioid Medications. Pain medicine (Malden, Mass). 2018;19(11). https://doi.org/10.1093/pm/pnx276

20.	Matthias MS, Johnson NL, Shields CG, Bair MJ, MacKie P, Huffman M, et al. “I'm Not Gonna Pull the Rug out From Under You”: Patient-Provider Communication About Opioid Tapering. The Journal of Pain. 2017;18(11):1365–73. https://doi.org/10.1016/j.jpain.2017.06.008

21.	Krebs E, Bergman A, Coffing J, Campbell S, Frankel R, Matthias M. Barriers to guideline-concordant opioid management in primary care--a qualitative study. The journal of pain. 2014 Nov;15(11). https://doi.org/10.1016/j.jpain.2014.08.006

22.	Cross AJ, Buchbinder R, Mathieson S, Bourne A, Maher CG, Lin C-WC, et al. Barriers and enablers to monitoring and deprescribing opioid analgesics for chronic non-cancer pain: a systematic review with qualitative evidence synthesis using the Theoretical Domains Framework. BMJ Quality & Safety. 2022–05–01;31(5). https://doi.org/10.1136/bmjqs-2021-014186

23.	Desveaux L, Saragosa M, Kithulegoda N, Ivers N. Understanding the behavioural determinants of opioid prescribing among family physicians: a qualitative study. BMC Fam Pract. 05/10/2019;20(1). https://doi.org/10.1186/s12875-019-0947-2

24.	Hamilton M, Mathieson S, Gnjidic D, Jansen J, Weir K, Shaheed C, et al. Barriers, facilitators, and resources to opioid deprescribing in primary care: experiences of general practitioners in Australia. Pain. 04/01/2022;163(4). https://doi.org/10.1097/j.pain.0000000000002340

25.	Langford A, Gnjidic D, Lin C, Bero L, Penm J, Blyth F, et al. Challenges of opioid deprescribing and factors to be considered in the development of opioid deprescribing guidelines: a qualitative analysis. BMJ quality & safety. 2021 Feb;30(2). https://doi.org/10.1136/bmjqs-2020-010881

26.	Anderson TS, Wang BX, Lindenberg JH, Herzig SJ, Berens DM, Schonberg MA. Perspectives on Using, Prescribing, and Deprescribing Opioids for Chronic Pain. JAMA Network Open. 2024/03/04;7(3). https://doi.org/10.1001/jamanetworkopen.2024.1342

27.	Noh H, Jeong H, Kan D, Zhang P, Lee LH. Older Adults’ Perspectives on Deprescribing and Non-Pharmacological Pain Management in the Deep South. J Appl Gerontol. 2025–8–16. https://doi.org/10.1177/07334648251411577

28.	Woodcock C, Cornwall N, Dikomitis L, Harrisson SA, White S, Helliwell T, et al. Designing a primary care pharmacist-led review for people treated with opioids for persistent pain: a multi-method qualitative study. BJGP Open. 2024 Aug 7;8(3). https://doi.org/10.3399/BJGPO.2023.0221

29.	De Sola H, Maquibar A, Failde I, Salazar A, Goicolea I. Living with opioids: A qualitative study with patients with chronic low back pain. Health Expect. 2020/10/01;23(5). https://doi.org/10.1111/hex.13089

30.	Ballard PJ, Arnold TJ, Vidrascu EM, Hernandez GC, Ozer E, Wolfson M, et al. Pathways to opioid use and implications for prevention: voices of young adults in recovery. Substance Abuse Treatment, Prevention, and Policy 2024 19:1. 2024–01–18;19(1). https://doi.org/10.1186/s13011-023-00584-5

31.	Tong S, Hochheimer C, Brooks E, Sabo R, Jiang V, Day T, et al. Chronic Opioid Prescribing in Primary Care: Factors and Perspectives. Ann Fam Med. 2019 May;17(3). https://doi.org/10.1370/afm.2357

32.	Deci EL, Ryan RM. The “What” and “Why” of Goal Pursuits: Human Needs and the Self-Determination of Behavior. Psychol Inq. 2000;11:227–68.

33.	Ryan R, Deci E. Self-determination theory and the facilitation of intrinsic motivation, social development, and well-being. The American psychologist. 2000;55(1). https://doi.org/10.1037//0003-066x.55.1.68

34.	Ryan RM, Deci EL. Self-determination theory: Basic psychological needs in motivation, development, and wellness: The Guilford Press.; 2017.

35.	Ntoumanis N, Ng JYY, Prestwich A, Quested E, Hancox JE, Thøgersen-Ntoumani C, et al. A meta-analysis of self-determination theory-informed intervention studies in the health domain: effects on motivation, health behavior, physical, and psychological health. Health Psychol Rev. 2021;15(2). https://doi.org/10.1080/17437199.2020.1718529

36.	Ryan RM, Patrick H, Deci EL, Williams GC. Facilitating health behaviour change and its maintenance: Interventions based on Self-Determination Theory. The European Health Psychologist. 2008;10.

37.	Patrick H, Williams GC. Self-determination theory: its application to health behavior and complementarity with motivational interviewing. Int J Behav Nutr Phys Act. 2012 Mar 2;9(1). https://doi.org/10.1186/1479-5868-9-18

38.	Henry SG, Paterniti DA, Feng B, Iosif A-M, Kravitz RL, Weinberg G, et al. Patients’ Experience With Opioid Tapering: A Conceptual Model With Recommendations for Clinicians. The Journal of Pain. 2019;20(2):181–91. https://doi.org/10.1016/j.jpain.2018.09.001

39.	Nichols VP, Toye F, Eldabe S, Sandhu HK, Underwood M, Seers K. Experiences of people taking opioid medication for chronic non-malignant pain: a qualitative evidence synthesis using meta-ethnography. BMJ Open. 2020 Feb 18;10(2). https://doi.org/10.1136/bmjopen-2019-032988

40.	Tong A, Sainsbury P, Craig J. Consolidated criteria for reporting qualitative research (COREQ): a 32-item checklist for interviews and focus groups. International journal for quality in health care : journal of the International Society for Quality in Health Care. 2007;19(6). https://doi.org/10.1093/intqhc/mzm042

41.	Sundhedsdatastyrelsen. MedicinForbrug - Indblik: Færre langtidsbrugere af opioider i 2017 [Internet]. 2018 [cited 2025 Jun 19]. Available from: https://sundhedsdatastyrelsen.dk/media/16184/MedicinForbrug_Indblik_Opioider_2017.pdf.

42.	Netværk for viden og kompetencer i almen praksis. Deltag i aktuelle almenmedicinske forskningsprojekter [Internet]. 2025 [2025]. Available from: https://www.vidap.dk/deltagiforskning.

43.	Nielsen SH, Søndergaard J, Andersen MKK, Pedersen LB. Predicting long-term treatment with short-acting opioids: the role of general practice consultations and sociodemographic factors. Fam Pract. 2026;43(2). https://doi.org/10.1093/fampra/cmag011

44.	Praktiserende Lægers Organisation. Honorarer og ydelser [Internet]. læger.dk; 2025 [cited 2025 24. Sep]. Available from: https://laeger.dk/foreninger/plo/overenskomsten-og-aftaler/honorarer.

45.	Malterud K, Siersma VD, Guassora AD, Kirsti Malterud VDS, Ann Dorrit Guassora. Sample Size in Qualitative Interview Studies. Qual Health Res. 2016–11;26(13). https://doi.org/10.1177/1049732315617444

46.	NVivo (version 14). Burlington (MA): Lumivero; 2023.

47.	Galbin A. An introduction to social constructionism. Social Research Reports. 2014;26:82–92. https://doi.org/10.5860/choice.33-3018

48.	Berger P, Luckmann T. The social construction of reality: A treatise in the sociology of knowledge. New York: Anchor Books; 1966.

49.	Braun V, Clarke V. Thematic Analysis - A Practical Guide: SAGE Publications Ltd; 2022.

50.	Braun V, Clarke V. Using thematic analysis in psychology. Qualitative Research in Psychology. 2006;3(2):77–101. https://doi.org/10.1191/1478088706qp063oa

51.	Vrijders B, Weinstein N, Paulmann S, Soenens B, Waterschoot J, Vansteenkiste M. Your prosody matters! The effect of controlling tone of voice on listeners’ experienced pressure, closeness, and intention to collaborate with the speaker. Motivation Science. 2025;11(1). https://doi.org/10.1037/mot0000357

52.	Leget D, LaCaille L, Hooker S, LaCaille R, Lauritsen M. Applying self-determination theory to internalized weight stigma and mental health correlates among young and middle adult women: A structural equation model. J Health Psychol. 2025 Feb;30(2). https://doi.org/10.1177/13591053241248283

53.	National Institute for Health and Care Excellence. Chronic pain (primary and secondary) in over 16s: assessment of all chronic pain and management of chronic primary pain. [Internet]. London, NICE Clinical Guideline No. NG193; 2021. Available from: https://www.nice.org.uk/guidance/ng193.

54.	Benintendi A, Kosakowski S, Lagisetty P, Larochelle M, Bohnert ASB, Bazzi AR. “I felt like I had a scarlet letter”: Recurring experiences of structural stigma surrounding opioid tapers among patients with chronic, non-cancer pain. Drug Alcohol Depend. 2021/05/01;222. https://doi.org/10.1016/j.drugalcdep.2021.108664

55.	Tsai AC, Kiang MV, Barnett ML, Beletsky L, Keyes KM, McGinty EE, et al. Stigma as a fundamental hindrance to the United States opioid overdose crisis response. PLoS Med. 2019 Nov 26;16(11). https://doi.org/10.1371/journal.pmed.1002969

56.	Cheetham A, Picco L, Barnett A, Lubman DI, Nielsen S. The Impact of Stigma on People with Opioid Use Disorder, Opioid Treatment, and Policy. Subst Abuse Rehabil. 2022 Jan 25;13. https://doi.org/10.2147/SAR.S304566

57.	Deriu V, Altavilla D, Adornetti I, Chiera A, Ferretti F. Narrative identity in addictive disorders: a conceptual review. Front Psychol. 2024 Jun 17;15. https://doi.org/10.3389/fpsyg.2024.1409217

58.	Stotts AL, Green C, Masuda A, Grabowski J, Wilson K, Northrup T, et al. A Stage I Pilot Study of Acceptance and Commitment Therapy for Methadone Detoxification. Drug Alcohol Depend. 2012 Mar 15;125(3). https://doi.org/10.1016/j.drugalcdep.2012.02.015
