# Supplementary material for: Barriers and motivators for opioid deprescribing among Danish adults receiving long-term treatment with short-acting opioids: an exploratory qualitative study in primary care informed by self-determination theory
Source: Scand J Prim Health Care. 2026 Aug 26;44(1):2721337. doi: 10.1080/02813432.2026.2721337 (PMC13523111; doi:10.1080/02813432.2026.2721337)
Supplement: Supplementary file 2 [file IPRI_A_2721337_SM1363.docx]

Appendix

**Barriers and motivators for opioid deprescribing among Danish adults receiving long-term treatment with short-acting opioids: An exploratory qualitative study in primary care informed by self-determination theory.**

Appendix A: COREQ (Consolidated criteria for Reporting Qualitative Research) Checklist	2

Appendix B: Semi structured interview guide	5

Appendix C: Reflections on the role of researcher	9

# Appendix A: COREQ (Consolidated criteria for Reporting Qualitative Research) Checklist

COREQ (COnsolidated criteria for REporting Qualitative research) Checklist

A checklist of items that should be included in reports of qualitative research. You must report the page number in your manuscript where you consider each of the items listed in this checklist. If you have not included this information, either revise your manuscript accordingly before submitting or note N/A.

| **Topic** | **Item No.** | **Guide Questions/Description** | **Reported on**  **Page No.** |
| --- | --- | --- | --- |
| **Domain 1: Research team and reﬂexivity** | | | |
| *Personal characteristics* | | | |
| Interviewer/facilitator | 1 | Which author/s conducted the interview or focus group? | Section 2.4 |
| Credentials | 2 | What were the researcher’s credentials? E.g. PhD, MD | Titlepage |
| Occupation | 3 | What was their occupation at the time of the study? | Appendix C |
| Gender | 4 | Was the researcher male or female? | Appendix C |
| Experience and training | 5 | What experience or training did the researcher have? | Appendix C |
| *Relationship with*  *participants* | | | |
| Relationship established | 6 | Was a relationship established prior to study commencement? | Section 2.2 |
| Participant knowledge of  the interviewer | 7 | What did the participants know about the researcher? e.g. personal goals, reasons for doing the research | Appendix C |
| Interviewer characteristics | 8 | What characteristics were reported about the interviewer/facilitator? e.g. Bias, assumptions, reasons and interests in the research topic | Appendix C |
| **Domain 2: Study design** | | | |
| *Theoretical framework* | | | |
| Methodological orientation and Theory | 9 | What methodological orientation was stated to underpin the study? e.g. grounded theory, discourse analysis, ethnography, phenomenology, content analysis | Section 2.5 |
| *Participant selection* | | | |
| Sampling | 10 | How were participants selected? e.g. purposive, convenience, consecutive, snowball | Section 2.4 |
| Method of approach | 11 | How were participants approached? e.g. face-to-face, telephone, mail, email | Section 2.2 |
| Sample size | 12 | How many participants were in the study? | Section 3 |
| Non-participation | 13 | How many people refused to participate or dropped out? Reasons? | Section 3 |
| *Setting* | | | |
| Setting of data collection | 14 | Where was the data collected? e.g. home, clinic, workplace | Section 3 |
| Presence of non-  participants | 15 | Was anyone else present besides the participants and researchers? | Section 3 |
| Description of sample | 16 | What are the important characteristics of the sample? e.g. demographic data, date | Section 3 |
| *Data collection* | | | |
| Interview guide | 17 | Were questions, prompts, guides provided by the authors? Was it pilot tested? | Section 2.3,  Appendix B |
| Repeat interviews | 18 | Were repeat interviews carried out? If yes, how many? | N/A |
| Audio/visual recording | 19 | Did the research use audio or visual recording to collect the data? | Section 2.4 |
| Field notes | 20 | Were ﬁeld notes made during and/or after the interview or focus group? | N/A |
| Duration | 21 | What was the duration of the interviews or focus group? | Section 3 |
| Data saturation | 22 | Was data saturation discussed? | Section 2.4 |
| Transcripts returned | 23 | Were transcripts returned to participants for comment and/or correction? | Section 2.4 |

| **Topic** | **Item No.** | **Guide Questions/Description** | **Reported on**  **Page No.** |
| --- | --- | --- | --- |
| **Domain 3: analysis and ﬁndings** | | | |
| *Data analysis* | | | |
| Number of data coders | 24 | How many data coders coded the data? | Section 2.5 |
| Description of the coding tree | 25 | Did authors provide a description of the coding tree? | N/A |
| Derivation of themes | 26 | Were themes identiﬁed in advance or derived from the data? | Section 2.5 |
| Software | 27 | What software, if applicable, was used to manage the data? | Section 2.4 |
| Participant checking | 28 | Did participants provide feedback on the ﬁndings? | N/A |
| *Reporting* | | | |
| Quotations presented | 29 | Were participant quotations presented to illustrate the themes/ﬁndings?  Was each quotation identiﬁed? e.g. participant number | Section 3.1.1, 3.1.2, 3.1.3, 3.2.1, 3.2.2, 3.3.1, and 3.3.2 |
| Data and ﬁndings consistent | 30 | Was there consistency between the data presented and the ﬁndings? | N/A |
| Clarity of major themes | 31 | Were major themes clearly presented in the ﬁndings? | Section 3, 3.1, 3.1.1, 3.1.2, 3.1.3, 3.2, 3.2.1, 3.2.2, 3.3, 3.3.1, and 3.3.2 |
| Clarity of minor themes | 32 | Is there a description of diverse cases or discussion of minor themes? | N/A |

Developed from: Tong A, Sainsbury P, Craig J. Consolidated criteria for reporting qualitative research (COREQ): a 32-item checklist for interviews and focus groups. *International Journal for Quality in Health Care*. 2007. Volume 19, Number 6: pp. 349 – 357

# Appendix B: Semi structured interview guide

**Section 1: Pain and reasons for opioid treatment**

| Questions |  | Probing questions |
| --- | --- | --- |
| Could you start by telling me a bit about how your pain started? |  | Where in your body do you experience pain?  How long ago did your pain start?  How did your pain affect your everyday life before you started on [your type of opioid]? |

**Section 2: Opioid treatment and its effects**

| Can you describe the process of how you started opioid treatment? |  | Why were you started on opioid treatment?  Can you tell me a bit about how you started opioid treatment?   - Who initiated the treatment? - What type of opioid? - What was the expected duration of treatment?   Have you tried different types of opioids? If yes, which ones?   - Why did you switch? |
| --- | --- | --- |
| Had you tried other types of pain treatment before you started on opioids? |  | Had you tried other types of medication for pain before you started on opioids?   - Which ones? - How effective were they?   Had you tried other measures or treatments for pain besides medication before you started on opioids? For example, physiotherapy?   - Which ones? - How effective were they? |
| Can you describe how opioids have affected your pain and your daily life? |  | How has [opioid type] affected your everyday life?  Do you feel that [opioid type] provides sufficient pain relief?   - If not, when do you experience pain? - How does the pain compare to when you were not taking [opioid type]?   Has the pain-relieving effect of [opioid type] changed over time?  In what ways has [opioid type] influenced your daily life? |
| Have you experienced any side effects or negative consequences from taking opioids? |  | Which ones?  Have they changed over time?  Were there any effects that surprised you?  Have you ever chosen not to take your medication because of side effects? |

**Section 3: Personal reflections on taking opioids**

| How do you feel about taking opioids? |  | Are there any feelings or thoughts that come up when you think about your use of [opioid type]?  Do you have any positive reflections on using [opioid type]?  Do you have any concerns about taking opioids?   - If yes, what concerns do you have?   Does it matter to you what others think about your opioid use?   - If yes, what thoughts do you have about this? - How does it affect you?   Have you talked to your GP about your concerns?   - If not, why not?   Do you talk with anyone other than your GP about taking opioids and the thoughts or concerns you may have?   - If yes, with whom? - If not, why not? |
| --- | --- | --- |

**Section 4: Thoughts on tapering and future challenges**

| Can you tell me about any experiences you have had with stopping or reducing your opioid use? |  | Have you tried to stop or reduce [opioid type] more than once?  Can you describe your experience(s)?  Whose initiative was it to stop or reduce the dosage? For example:   - Your GP’s - Your own - A relative’s - A shared decision   Was the treatment reduced or stopped completely?  What was the reason for reducing or stopping the treatment?  How did tapering or discontinuation affect your everyday life?  How did you experience the support you received during the tapering process?  Was anything particularly helpful? And why? For example:   - Contact with your GP - Nurse - Family - Other   Why was the treatment started again?  How did this (these) experience(s) influence your willingness to attempt reduction or discontinuation again?  *If the participant has attempted tapering more than once:*  Have your experiences influenced how you handled later attempts at tapering or discontinuation?  *If the participant has not attempted tapering more than once:*  Do your experiences influence how you would handle any future attempts at tapering or discontinuation? |
| --- | --- | --- |
| Can you tell me about any thoughts you have had regarding stopping or continuing opioid use in the future? |  | If yes, what thoughts have you had?  What are your thoughts about the possibility of stopping or reducing your opioid use?  Has anything helped you consider the possibility of stopping? For example:   - Personal experiences - The internet - Your GP - Friends - Other people’s experiences   Have you ever felt external pressure to stop or reduce your treatment?   - If yes, from whom? - If yes, how did this affect your willingness to stop or reduce the treatment?   Have you talked to your GP about your thoughts on reduction or discontinuation?   - If not, why not? |

*Table note: GP, General practitioner*

# Appendix C: Reflections on the role of researcher

As the interviewer and primary analyst, my professional background inevitably shaped both data production and interpretation. I am a female PhD student trained in basic medicine, quality improvements, patient safety, and health economics, but without a clinical specialization. This meant that I approached the interviews without a physician’s authority, which may have created a relational dynamic where participants felt less compelled to justify their choices in clinical terms. Several patients appeared to receive the interview as an open and non-judgmental space, and some explicitly expressed that they found it to be a positive and relieving conversation. It is possible that this dynamic facilitated greater openness on sensitive issues such as ambivalence, stigma, or strategies for managing use. Participants had no knowledge about me as a researcher, except knowing my educational background and current position.

At the same time, my non-clinical background required me to consider how the findings would resonate within clinical contexts. While this process added interpretive depth, it also reflects that my lens was not that of a prescribing clinician. Also, my lack of previous profound training in conducting qualitative studies undoubtedly influenced the interview. Even though I tried my best not to ask leading questions, I might have used such in situations where a more experienced researcher would not. In addition, my preconceptions may have influenced both the conduct of interviews and the analysis. For instance, when asking about external pressures, I implicitly assumed that these would primarily concern tapering. While this assumption was often confirmed, some participants also described pressure to increase their dose. This nuance may have been underrepresented, as my prior assumptions shaped both questioning and interpretation.

In line with reflexive thematic analysis, it is acknowledged that complete neutrality is neither possible nor desirable. Rather, knowledge is produced through the interaction between the researcher and the participants, and my professional and personal positioning has undoubtedly shaped the narratives presented. Recognizing these dynamics is crucial for clarifying both the possibilities and limitations of the study. This reflexive stance also aligns with the social constructionist epistemological approach underpinning the study, in which data are not seen as transparent accounts of reality, but as co-constructed meanings shaped by discourses, contexts, and the researcher’s interpretive role.
